# Pennsylvania Hospital

**Published:** 1864-04

**Authors:** 


PENNSYLVANIA HOSPITAL.—Dr. Joseph Pancoast has resigned his situation as one of the Surgeons of this Institution, and Dr. Thomas George Morton has been selected in his place.

